# RETRACTED: Chen, N.; Zhang, B. IAV Antagonizes Host Innate Immunity by Weakening the LncRNA-LRIR2-Mediated Antiviral Functions. *Biology* 2024, *13*, 998

**DOI:** 10.3390/biology14080960

**Published:** 2025-07-31

**Authors:** Na Chen, Baoge Zhang

**Affiliations:** College of Veterinary Medicine, Nanjing Agricultural University, Nanjing 210095, China; 2021207051@stu.njau.edu.cn

The journal retracts the article titled “IAV Antagonizes Host Innate Immunity by Weakening the LncRNA-LRIR2-Mediated Antiviral Functions” [1], cited above.

Following publication, the authors contacted the Editorial Office to raise concerns regarding unauthorized use of propriety data presented within this article [1].

Adhering to our standard policy, an investigation was conducted that confirmed with the Nanjing Agricultural University and the authors that appropriate permission to publish the underlying data had not been obtained. As a result, the Editorial Office, the Editorial Board, and the authors have decided to retract this article as per MDPI’s retraction policy (https://www.mdpi.com/ethics#_bookmark30).

This retraction was approved by the Editor-in-Chief of the *Biology* journal.

The authors agreed to this retraction.
